# DISC1 Regulates Primary Cilia That Display Specific Dopamine Receptors

**DOI:** 10.1371/journal.pone.0010902

**Published:** 2010-05-28

**Authors:** Aaron Marley, Mark von Zastrow

**Affiliations:** Departments of Psychiatry and Cellular & Molecular Pharmacology, University of California San Francisco, San Francisco, California, United States of America; Mental Health Research Institute of Victoria, Australia

## Abstract

**Background:**

Mutations in the DISC1 gene are strongly associated with major psychiatric syndromes such as schizophrenia. DISC1 encodes a cytoplasmic protein with many potential interaction partners, but its cellular functions remain poorly understood. We identified a role of DISC1 in the cell biology of primary cilia that display disease-relevant dopamine receptors.

**Methodology/Principal Findings:**

A GFP-tagged DISC1 construct expressed in NIH3T3 cells and rat striatal neurons localized near the base of primary cilia. RNAi-mediated knockdown of endogenous DISC1 resulted in a marked reduction in the number of cells expressing a primary cilium. FLAG-tagged versions of the cloned human D1, D2 and D5 dopamine receptors concentrated highly on the ciliary surface, and this reflects a specific targeting mechanism specific because D3 and D4 receptors localized to the plasma membrane but were not concentrated on cilia.

**Conclusions/Significance:**

These results identify a role of DISC1 in regulating the formation and/or maintenance of primary cilia, and establish subtype-specific targeting of dopamine receptors to the ciliary surface. Our findings provide new insight to receptor cell biology and suggest a relationship between DISC1 and neural dopamine signaling.

## Introduction

The disrupted-in-schizophrenia (DISC) genetic locus was discovered as a balanced translocation segregating as a strong risk factor for major psychiatric syndromes including schizophrenia, bipolar disorder and major depression [1], [2]. Subsequent studies have consistently verified the importance of one of the genes disrupted by this translocation (DISC1). DISC1 has been reproducibly linked to psychiatric disorders involving impairment of cognitive function, particularly schizophrenia [3]. Further, DISC1-mutant mice exhibit anatomical and behavioral deficits that are generally consistent with DISC1-associated psychopathology observed in humans [4].

Given these compelling genetic data, critical challenges moving forward are to elucidate the cellular basis of DISC1 function under normal conditions, and to determine fundamental consequences of DISC1 disruption. These are areas of intensive current investigation, and exciting progress has already been made. Summarized very briefly, the present data suggest that DISC1 functions in multiple cellular processes affecting neural development and synaptic structure or activity [5], [6]. Precise cellular mechanisms underlying these diverse effects, however, remain largely mysterious. Based on a serendipitous observation, we tested the hypothesis that DISC1 affects the cell biology of primary cilia. Our results support such a link, and reveal specific ciliary targeting of dopamine receptors implicated in schizophrenia.

## Results

### DISC1-GFP localizes near the base of primary cilia

**Figure 1:**
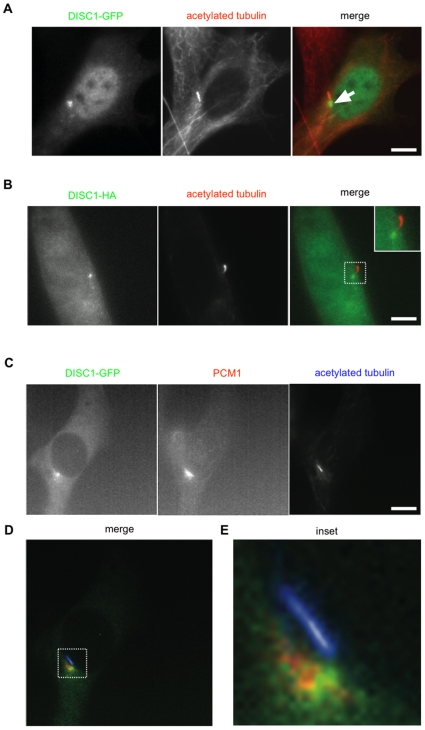
Localization of DISC1-GFP near the base of primary cilia. (**A**) NIH3T3 cells transfected with DISC1-GFP were fixed and immunolabeled for acetylated tubulin to mark primary cilia. An example of such a double-labeled cell is shown, with DISC1-GFP in green and acetylated tubulin in red in the merged image. Arrow indicates indicates the concentration of DISC1-GFP observed near the ciliary base. (**B**) The same experiment conducted using DISC1-HA. (**C**) Triple localization of DISC1-GFP, endogenous PCM1, and acetylated tubulin verifying localization of DISC1 in a centrosomal region near the ciliary base. (**D**) Merged image from the triple localization with DISC1-GFP in green, PCM1 in red, and acetylated tubulin in blue. (**E**) The region indicated in panel D displayed at higher magnification. Scale bar, 10 µm.

In the course of carrying out a distinct series of experiments, we examined the localization of a GFP-tagged human DISC1 fusion construct expressed in transfected NIH3T3 cells. Punctate concentrations of GFP-DISC1 were often observed near the nucleus, and near the base of primary cilia marked by acetylated tubulin (Fig. 1A). We observed a similar distribution of DISC1 tagged with a distinct HA epitope rather than GFP (Fig. 1B). This distribution is consistent with association of DISC1 with centrosomal components, as reported previously [7], [8]. Triple localization of DISC1 (green) with the pericentriolar protein PCM1 (red) and acetylated tubulin marking cilia (blue) verified proximity of DISC1 both to pericentriolar components and the primary cilium (Fig. 1C and D). Because a number of centrosome-localized proteins affect the formation or regulation of primary cilia [9], we wondered if DISC1 plays any role in ciliary biology.

### Depleting endogenous DISC1 results in loss of primary cilia

**Figure 2:**
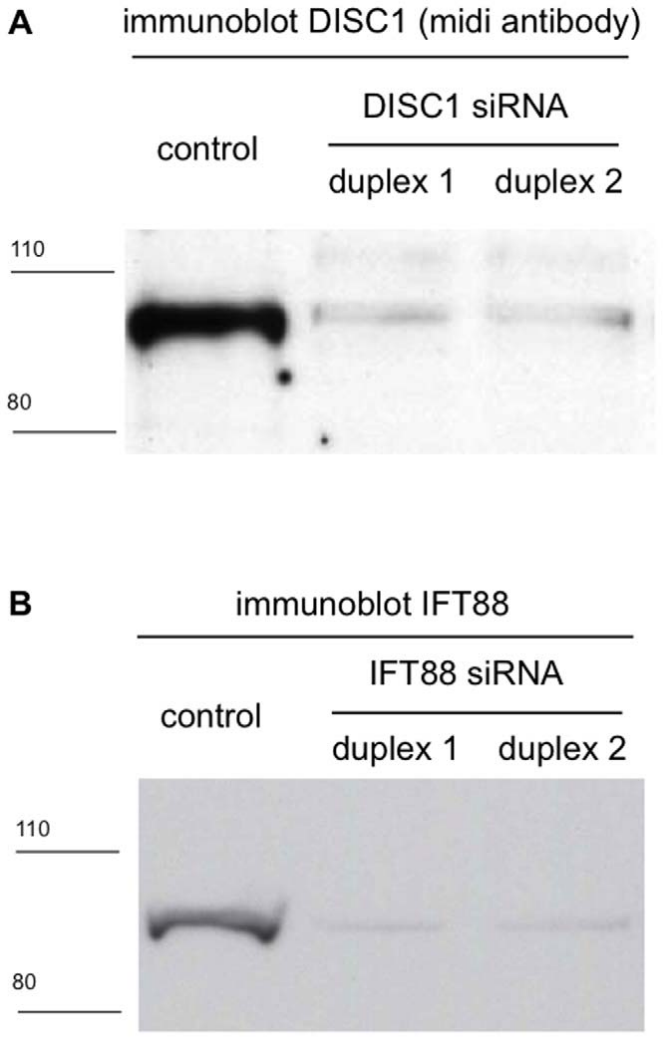
Depletion of endogenous DISC1 and IFT88 by RNA interference. NIH3T3 cells were transfected with the indicated siRNA duplexes (sequences are listed in *Materials and Method*s). Total cell extracts were prepared 72 hours later, and levels of DISC1 (**A**) or IFT88 (**B**) protein were assessed by immunoblot using antibodies recognizing the endogenous proteins. Representative immunoblots are shown. Densitometric scanning across multiple experiments verified >80% depletion of both proteins by the respective siRNA duplexes.

To investigate this question, we first asked if depleting endogenous DISC1 protein affected cilia number in NIH3T3 cells. To accomplish this, we identified two independent siRNA duplexes producing reliable knockdown of endogenous DISC1 (Fig. 2A; scanning densitometry estimated >80% reduction in immunoreactive DISC1 protein). As a positive control, we verified two RNA duplexes depleting the essential ciliary protein IFT88 (Fig. 2B).

**Figure 3:**
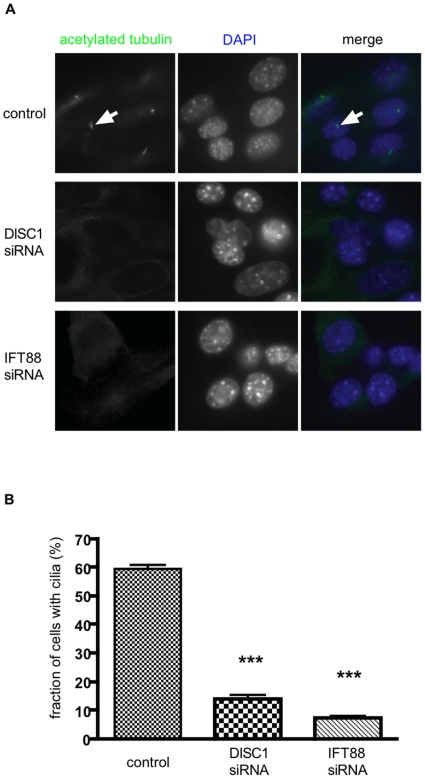
DISC1 knockdown reduces cilia number. (**A**) NIH3T3 cells were transfected with the indicated RNA duplexes and then stained with anti-acetylated tubulin (to mark primary cilia, left panels) and DAPI (to mark nuclei, middle panels). Merged images (right panels) show acetylated tubulin and DAPI staining in green and blue, respectively. Cilia were prominently observed in the majority of cells transfected with control (non-silencing) RNA duplexes (top row of images, an example is indicated by arrow). In cells transfected with either siRNA targeting DISC1, the vast majority of cells did not project a detectable primary cilium. Depleting IFT88 (bottom row of images) produced a similar effect. (**B**) Quantification of cilia loss by counting the number of cells extending a primary cilium, as marked by acetylated tubulin immunostaining, observed in blinded analysis of the indicated populations of siRNA-transfected cells. Bars represent the mean fraction of cells with a visible cilium, averaged across 5 experiments counting ≥250 cells/condition in each. Error bars represent the s.e.m. calculated across the experiments (***, p<0.0001).

**Figure 4:**
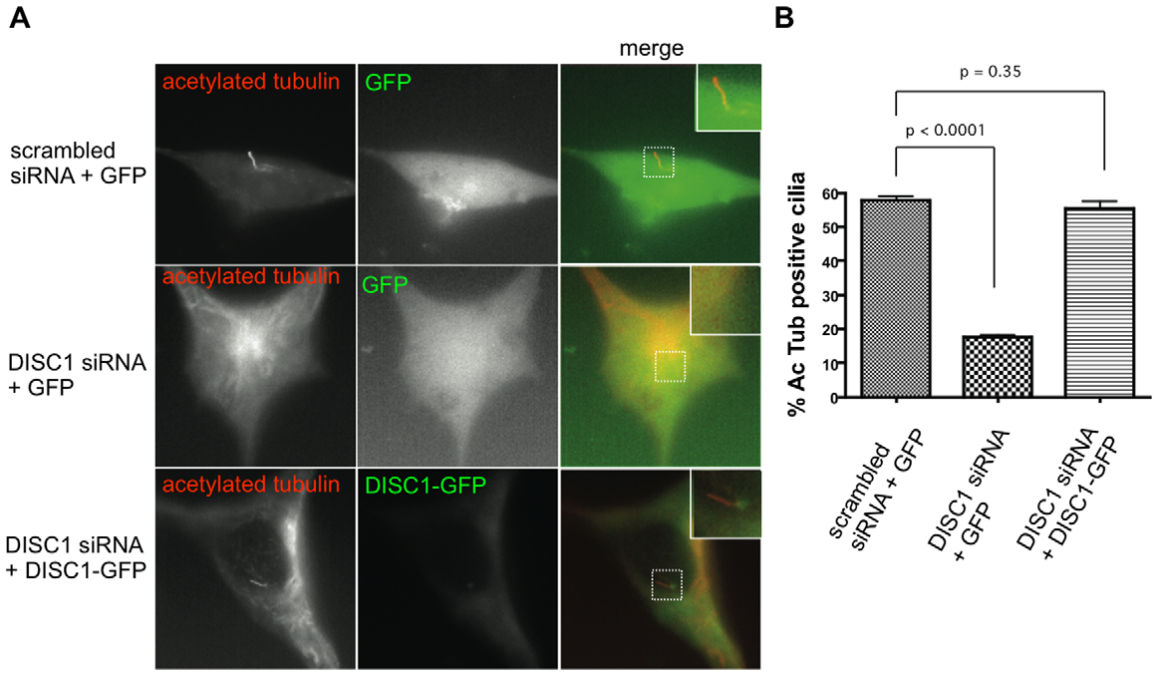
Rescue of the DISC1 knockdown phenotype by human-derived DISC1-GFP. NIH3T3 cells were transfected with siRNA targeting endogenous murine DISC1 and transduced with lentiviral particles encoding human-derived DISC1-GFP lacking the target sequence. (**A**) Example images from the rescue experiment. Top panels show the negative control condition, in which cells were transfected with scrambled (non-silencing) siRNA and infected with a lentivirus encoding GFP. Middle panels show the knockdown condition, in which cells were transfected with DISC1 siRNA and infected with lentivirus encoding GFP. Bottom paneles show the rescue condition, in which cells were transfected with DISC1 siRNA and infected with lentivirus encoding the siRNA-resistant DISC1-GFP construct. Left panels show acetylated tubulin immunoreactivity, middle panels show GFP fluorescence, and right panels show the merge image with inset as indicated by box. (**B**) Quantification of the rescue effect by cilia count (n = 4 experiments).

Using the nuclear stain DAPI (blue) to identify individual cells, and acetylated tubulin (green) to mark cilia, we observed primary cilia on the majority of cells transfected with control (non-silencing) siRNA duplex (Fig. 3A, top row of panels; a particular example is indicated by arrow). In cells transfected with DISC1 siRNA, in contrast, we rarely observed a detectable cilium (Fig. 3A, middle row). This effect of DISC1 depletion was observed using both of the siRNA duplexes silencing DISC1. This effect was qualitatively similar to that of knocking down IFT88 (Fig. 3A, bottom row), whose depletion is already known to prevent cilia formation [10], [11]. We verified these results quantitatively by counting cilia-bearing cells across multiple experiments (Fig. 3B). To test specificity, we attempted to rescue this phenotype using a lentiviral vector encoding a human-derived DISC1-GFP construct that is resistant to knockdown by the rodent-specific duplexes (see *Materials and Methods*). Primary cilia were observed in the majority of knockdown cells following re-expression of DISC1-GFP (Fig. 4A), and quantification across multiple experiments indicated essentially complete rescue relative to the scrambled siRNA control (Fig. 4B). Together, these results suggest that DISC1 plays an important role in the normal formation or maintenance of primary cilia in this model cell system.

### A subset of dopamine receptors concentrate on the surface of primary cilia

Cilia are known to concentrate a variety of signaling receptors and mediators, supporting the general view that they function as a specialized signaling domain of the plasma membrane [12]. With this in mind, we asked if particular signaling receptors already implicated in schizophrenia might be located there. We focused on dopamine receptors, a subfamily of seven-transmembrane G protein-coupled receptors (GPCRs) whose significance to schizophrenia is firmly established. D2-class dopamine receptors represent major targets of both typical and atypical antipsychotics, have been linked to schizophrenia in human genetic studies, and are implicated most strongly in the so-called ‘positive’ symptoms of schizophrenia such as hallucinations [13], [14]. A correspondence between D2 receptor occupancy and antipsychotic effects has been convincingly verified in human neuroimaging studies [15]. D1-class receptors are implicated in cognitive deficits observed in schizophrenic patients [16], and may be related to a distinct cluster of ‘negative’ symptoms including social withdrawal [17], [18].

To investigate a potential link between DISC1-regulated cilia and dopamine receptors, we used epitope tagging to localize individual members of this receptor family relative to primary cilia marked by acetylated tubulin. In order to specifically examine dopamine receptors present in the plasma membrane, we engineered a Flag-epitope tag into the amino-terminal extracellular domain of each receptor and specifically decorated surface-exposed receptors by incubating intact cells with anti-Flag antibody. As a negative control, we expressed a Flag-tagged version of the human transferrin receptor (Flag-TfnR), a well characterized nutrient-uptake receptor that is present in the plasma membrane but is not known to localize to cilia [19].

**Figure 5:**
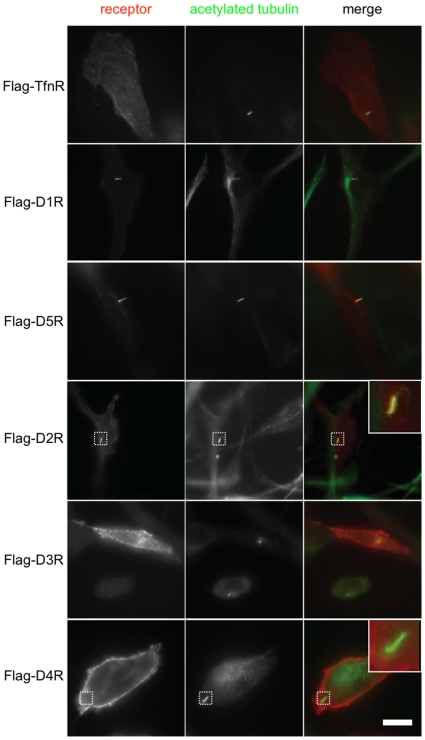
Subtype-specific localization of dopamine receptors to the primary cilium. NIH3T3 cells were transfected with expression construct encoding the indicated Flag-tagged dopamine receptor or transferrin receptor. 48 hours after transfection, cells were fixed and receptors present in the plasma membrane were selectively labeled by incubating non-permeabilized cells in the presence of anti-Flag antibody, and cells were then permeabilized to immunolabel acetylated tubulin. Each row of images shows representative cells expressing the indicated Flag-tagged receptor construct. The merged image in each row shows surface receptor immunoreactivity in red and acetylated tubulin in green to mark the primary cilium. Insets show the indicated region at higher magnification, scale bar corresponds to 10 µm.

As expected, Flag-TfnRs localized throughout the plasma membrane of NIH3T3 cells but were not detected on cilia (Fig. 5, top row of panels). In contrast both D1-class dopamine receptors (Flag-D1R and Flag-D5R), while also distributed throughout the plasma membrane, were prominently concentrated on the ciliary surface relative to the surrounding plasma membrane (rows 2 and 3 from top). One of the D2-class dopamine receptors (Flag-D2R) also concentrated prominently on the surface of cilia (row 4, a higher magnification view is shown in inset). This localization was specific for a subset of dopamine receptors because the closely related D2-class dopamine receptor, Flag-D4R, was diffusely localized in the plasma membrane but not detected on cilia (row 5 and inset). Another D2-class dopamine receptor, Flag-D3R, was also not observed on cilia although, in some cells, Flag-D3R concentrated in regions of the plasma membrane near the ciliary base (row 6).

**Figure 6:**
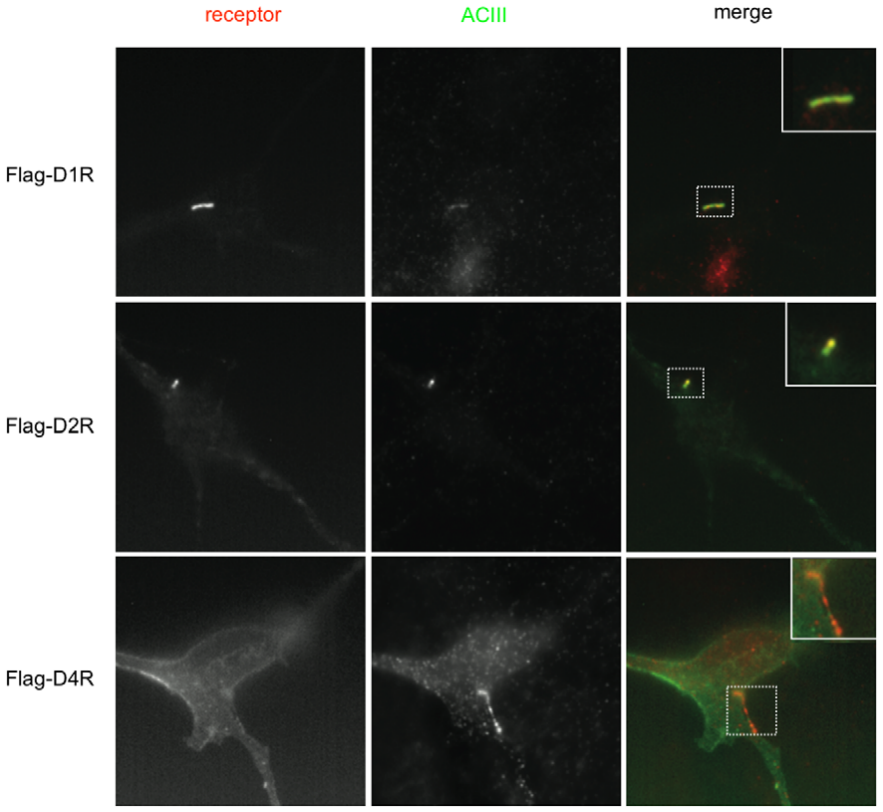
Subtype-selective localization of dopamine receptors to cilia in striatal neurons. Examples of ciliary localization of Flag-D1R and Flag-D2R, but not Flag-D4R, in primary striatal neurons. The left panels indicate surface Flag immunoreactivity marking receptors present in the plasma membrane. Middle panels indicate ACIII immunoreactivity marking cilia. The merged image is shown in the right panels, with the region of cell body containing the cilium displayed at higher magnification in the inset.

We next asked if these observations pertain to a physiologically relevant population of CNS-derived neurons. We focused on striatal medium spiny neurons because they express various dopamine receptors endogenously [20], and because a significant fraction of these neurons are ciliated in the intact brain [21], [22]. We examined the surface distribution of Flag-tagged versions of the cloned D1, D2 and D4 receptors when expressed in primary cultures of neurons dissociated from rat striatum, and compared the receptor localization observed to that of adenylyl cyclase type III (ACIII), shown previously to be a useful ciliary marker in the CNS [22]. Flag-D1Rs localized prominently to ACIII-positive cilia (Fig. 6, top set of panels; higher magnification of the relevant region is shown in inset), as did Flag-D2Rs (Fig. 6, middle row of panels). Flag-D4Rs, in contrast, were distributed elsewhere in the plasma membrane but were not detected on cilia (Fig. 6, bottom row of panels). This pattern of dopamine receptor localization mirrored that observed in NIH3T3 cells, suggesting the operation of a conserved targeting mechanism and establishing subtype-selective localization of dopamine receptors to the primary cilium in physiologically relevant neurons.

**Figure 7:**
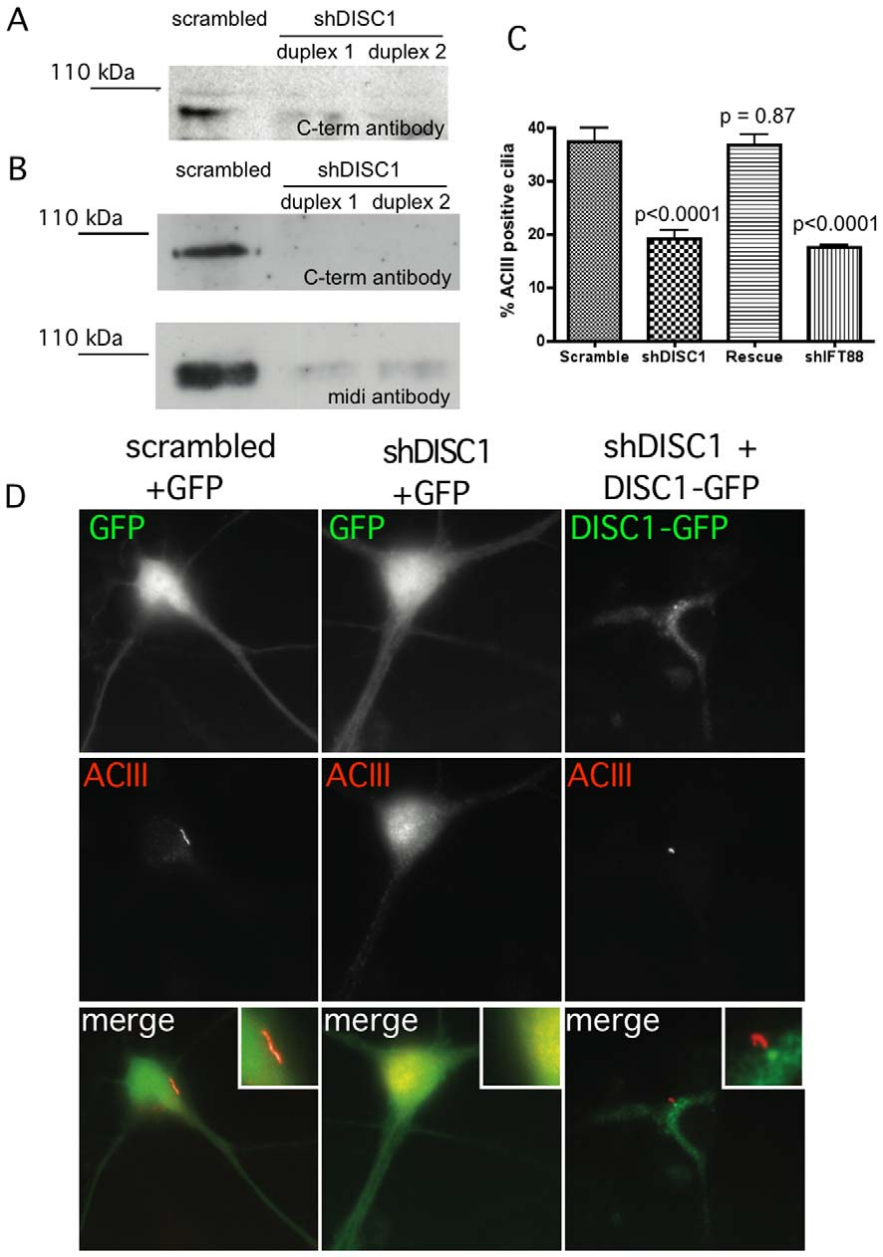
DISC1 knockdown and rescue in striatal neurons. (**A**) Example immunoblot showing depletion of endogenous DISC1 detected in PC12 cells using the C-terminal antibody (see *Materials and Methods*). The immunoreactive band corresponding to the major full length DISC1 species is shown. (**B**) Example of DISC1 depletion in striatal cultures, detected using the C-terminal (C-term) antibody and verified using the midi antibody, as indicated. (**C**) Quantification of knockdown and rescue phenotypes, based on count of cilia number compiled from 8 independent experiments. (**D**) Examples of the phenotypes observed in the indicated conditions (indicated above each column). The rescue condition (right column of images) shows the localization of DISC1-GFP rather than GFP. Insets indicate relevant regions of the cell body at higher magnification.

To further explore the potential applicability of our findings to the CNS, we next asked if cilia present on striatal neurons are sensitive to DISC1 depletion. To do so we used lentiviral expression of shRNA to knock down endogenous DISC1 in neuronal cultures. We first screened for effective shRNAs in rat-derived PC12 cells (Fig. 7A) and, after choosing two duplexes that produced reliable knockdown in this system, verified depletion of endogenous DISC1 in primary rat striatal neurons with two independent antibodies recognizing distinct portions of the DISC1 protein (Fig. 7B; details of each antibody are provided in *Materials and Methods*). We then used these tools to assess effects of DISC1 knockdown on ciliation. ACIII-marked cilia were observed on ∼40% of control striatal neurons, transduced with virus expressing the scrambled (non-silencing) duplex. DISC1 depletion significantly reduced the fraction of neurons expressing a visible cilium, and this effect was comparable in magnitude to that produced by shRNA targeting IFT88. Further, the effect of DISC1 knockdown in striatal neurons, as in NIH3T3 cells, could be rescued by expression of human-derived (shRNA-resistant) DISC1-GFP (Fig. 7C). Representative images from this experiment are shown in Fig. 7D. In the left and middle columns, control and knockdown conditions are shown using soluble GFP to mark the entire neuron. In the rescue condition (right column of panels), the green channel displays recombinant DISC1-GFP fluorescence, verifying localization of the rescue construct near the ciliary base of neurons.

## Discussion

The present results identify a role of DISC1 in regulating primary cilia, and show that dopamine receptors exhibit subtype-selective localization to these structures. We established both observations in a well-established non-neural cell model and in physiologically relevant CNS-derived neurons that are ciliated *in vivo*. Accordingly, our results provide new insight to the basic cell biology of both DISC1 and dopamine receptors. They also suggest the possibility that primary cilia may represent a fundamental organizing principle linking pathological mutations of DISC1 to the development and/or expression of major psychiatric disorders.

We are not aware of previous evidence linking DISC1 functionally to primary cilia, but the present findings are broadly consistent with biochemical data indicating that DISC1 binds to and exhibits similar localization as various centrosome-associated proteins [7], [23], [24], [25]. This includes Bbs4, an essential component of primary cilia that causes a ciliopathy (Bardet-Biedl syndrome) when disrupted [8], [26], and PCM1 as shown here. We are also not aware of previous evidence linking dopamine receptors to cilia. Ciliary localization of a number of other seven-transmembrane signaling proteins has been reported, however, including conventional GPCRs such as MCHR1 melanocortin receptors [27], 5HT6 serotonin receptors [28], SSTR3 somatostatin receptors [29] as well as more distantly related non-conventional seven-transmembrane proteins such as Smoothened [30]. The present results add to the number of conventional GPCRs that localize to cilia, and establish subtype-selective ciliary localization of particular dopamine receptors that have been genetically associated with schizophrenia (D2 receptors) or regulate disease-related features (D1 and D2 receptors).

These findings raise interesting mechanistic questions for future study. First, how does DISC1 depletion affect primary cilia? DISC1 did not localize to the cilium *per se* but concentrated near the ciliary base, consistent with previous data linking DISC1 to various centrosome-associated proteins. This suggests that DISC1 does not affect cilia directly but, instead, likely functions as an indirect regulator of cilia formation or maintenance. The biochemical mechanism of this proposed regulation is presently unknown. We note that the centrosome is a complex structure, including dynamic associated components such as centriolar satellites [31], [32]. The present data regarding DISC1 localization compared to PCM1 and acetylated tubulin suggests that DISC1 is optimally situated to interact both with centriolar and ciliary components. Second, what is the mechanism by which specific dopamine receptors localize to cilia? The current data indicate that individual members of the dopamine receptor family, which share extensive structural homology, differ significantly in their ability to localize to cilia. This was clear from the remarkable selectivity in ciliary targeting of D2 compared to D4 receptors, both in NIH3T3 cells and striatal neurons. A degenerate ciliary targeting motif, AxA/SxQ, was identified previously in the third cytoplasmic loop of several GPCRs [27]. We were unable to identify any cytoplasmic sequence matching this consensus in the cilia-targeted dopamine receptors examined in the present study. The most similar sequence that we could find is AKNCQ, present in the D1 dopamine receptor. This sequence is not conserved in either D2 or D5 receptors, even though these receptors also localize prominently to cilia. We also found that mutating the glutamine residue in this sequence to phenylalanine (Q242F), a substitution used to disrupt two such motifs identified previously in the SSTR3 receptor[27], did not detectably impair ciliary localization of Flag-D1Rs (data not shown). Thus, our results suggest the existence of additional structural determinant(s) determining subtype-specific localization of dopamine receptors to cilia. Another proposed mechanism of ciliary targeting is by association of receptors with β-arrestins (also called non-visual arrestins) that exhibit centrosomal localization [33], [34]. Previous studies suggest that D2Rs interact with arrestins relatively weakly [35] and none of the dopamine receptors are known to bind arrestins to a significant degree in their non-activated state [36], the condition examined in the presence study. Thus, our results suggest that this alternate mechanism is also not sufficient to fully explain the observed ciliary localization of dopamine receptors. We also note that D3 dopamine receptors, although not clearly observed on cilia, often localized near the cilia base. This suggests, further, that dopamine receptors may undergo cilary targeting by a multi-step mechanism involving more than one receptor-selective step.

Our results also raise a number of interesting physiological questions. Foremost among them are to determine whether DISC1 regulates primary cilia in the developing or adult brain, and to define the functional consequence(s) of subtype-specific localization of dopamine receptors to neuronal cilia. The role of primary cilia in the cell biology of postnatal neurons is poorly understood, but the existence of such structures has been recognized for some time [21], [22], [37], [38]. It is increasingly clear that primary cilia fundamentally organize various cellular signaling processes, including those emanating from conventional and atypical seven-transmembrane receptors [9], [12], [30], [39]. The present results add to the accumulating evidence for ciliary targeting of conventional GPCRs, and do so for dopamine receptors whose activity (or dysregulation of activity) is implicated in the expression of schizophrenic symptoms. Another important question for future study, given that present results are limited to depletion of wild type DISC1, is whether disease-associated mutations in DISC1 affect the structure or function of cilia. As a number of other disease-linked genes, such as PCM1 discussed above, localize near the ciliary base [9], [13] it remains an open question how pathological mutations affect their organization or function.

The ultimate objective of this line of investigation is to elucidate the cell biological underpinnings of complex neuropsychiatric disorders, using advances in human disease genetics as a guide. We believe that the results described here, establishing a previously unrecognized role of DISC1 in regulating primary cilia, and revealing ciliary localization of particular schizophrenia-relevant dopaminergic receptors, represent early progress toward this challenging and important goal.

## Materials and Methods

### cDNA constructs and cell culture

Human KIAA0457 (DISC1 long variant) was obtained from Kazusa (RIKEN). We generated a fluorescently tagged version of this construct by adding NheI and AgeI sites at the 5′ and 3′ ends, respectively, of the coding sequence by PCR amplification. The PCR product was then ligated in-frame to a 3′ sequence encoding enhanced GFP in the pIREShyg3 backbone (Clontech). FLAG-tagged versions of the human D1 and D2 (long isoform) dopamine receptors have been previously described [35]. cDNA encoding the human D4 dopamine receptor (D4.7 isoform) was a gift of Dr. Hubert Van Tol. cDNAs encoding human D3 and D5 receptors were obtained from the Missouri cDNA Resource Center (www.cdna.org). Tagged receptor constructs were cloned into pcDNA3 (Invitrogen) for expression in NIH3T3 cells and into pCAGGS [40] for expression in striatal neurons.

NIH3T3 cells (ATCC, Manassas, VA) were maintained in Dulbecco's modified Eagle's medium supplemented with 10% fetal calf serum (University of California, San Francisco, Cell Culture Facility). The fraction of ciliated cells declined with extended passaging, so all experiments were carried out using early-passage cells within 30 days of thaw. We utilized Effectene (Qiagen) to transiently transfect NIH3T3 cells with the cells the indicated tagged receptors constructs and examined 3 days after transfection or viral transduction (see below).

Dissociated striatal neurons were cultured from embryonic day 17–18 Sprague Dawley rat embryos. The striatum (caudate–putamen and nucleus accumbens) was dissected based on the criteria of Ventimiglia and Lindsay (1998) [41]. Upon dissection, tissue was dissociated in 1× trypsin/EDTA solution (Invitrogen) for 15 min before 1 ml of trypsin inhibitor was added for 5 min at room temperature. The suspension was triturated in DMEM plus 10% fetal calf serum (FCS; Invitrogen) using a glass pipette. Cells were plated on coverslips previously etched with 70% nitric acid and rinsed over 2–3 days, then coated with poly-L-lysine-coated (1 mg/ml in 0.1 M sodium borate buffer, pH 8.5) overnight, dried and washed extensively. Media on the cells was replaced with Gibco Neurobasal media (Invitrogen) supplemented with B27 (Gibco) and L-glutamine 24 h after plating. For tagged receptor expression, neurons were transfected using Lipofectamine (Invitrogen) at 5–7 DIV and examined 2 days later. Primary dissociated neurons were infected with lentiviral particles 1–2 DIV and examined 10 days later.

### Knockdown and recombinant protein expression

For knockdown of endogenous DISC1 in NIH3T3 cells, the following siRNA duplexes were obtained from Qiagen:

siRNA#1 (mM_DISC1_4): r(CACGGAGACCAGGCUACAUGA)

siRNA#2 (mM_DISC1_2): r(CAGCUGGAGGUCACUUCCUUA)

For knockdown of IFT88 in NIH3T3 cells we used the following siRNA duplexes, also obtained from Qiagen:

siRNA#1 (mM_IFT88_1), r(AAGGCAUUAGAUACUUAUAAA)dTdT

siRNA#2 (mM_IFT88_4)r(UUGGAGCUUAUUACAUUGAUA)dTdT.

The scrambled control sequence used was r(AAU UCU CCG AAC GUG UCA CG)dT

Duplexes were transfected using Lipofectamine RNAi-max (Invitrogen) using the optimized protocol provided by the manufacturer for NIH3T3 cells. In all experiments reagent amounts were scaled according to surface area of the specific culture dishes used, based on the optimized protocol listed for 24-well plates. Experiments were conducted 3 days after siRNA transfection without starvation.

For knockdown of endogenous DISC1 in neurons, short hairpin sequences were cloned as DNA oligos into pLKO.1 with AgeI/EcoRI, then shuttled into the dual short hairpin GFP expression vector pHUGW-GFP [42], [43].

shRNA DISC1 #1: GGCTACATGAGAAGCACAG

shRNA DISC1 #2: CAGCTGGAGGTCACTTCCTT

shRNA DISC1#1 is a previously validated sequence which has been shown to target mouse DISC1 [44]. The identical target sequence is present in rat DISC1.

For knockdown of endogenous IFT88 in neurons,

shRNA IFT88 #1: GCCCTCAGATAGAAAGACCAA

shRNA IFT88 #2: GCAGGAAGACTGAAAGTGAAT

The scrambled control sequence used was TCCTAAGGTTAAGTCGCCCTCT

For recombinant protein re-expression in both NIH3T3 cells and cultured neurons, DISC1-GFP was mutated by site directed mutagenesis to C GGGTATATGC in order to assure resistance to knockdown by rodent-targeted siRNA#1 and sh#1duplex (the duplexes used in rescue experiments; underlined residues show synonymous mutations introduced). This construct was then cloned by PCR into the pHUGW vector downstream of the Ub promoter using the restriction enzyme sites XbaI/AgeI.

Lentiviruses were generated from the constructs described above in fresh HEK293FT cells (Invitrogen) cultured in 10 cm dishes containing DMEM-H21, 10% FBS, 4 mM L-Glutamine, 1 mM MEM sodium Pyruvate, 0.1 mM MEM Non-Essential Amino Acids, 1% penicillin-streptomycin, and 500 ug/ml G418. Cells were transfected with pLKO.0 or pHUGW and the packaging vectors psPAX2, pVSV-G, at a ratio of 2/2/1 respectively, utilizing Lipofectamine 2000 (Invitrogen) as tranfection reagent. Medium was changed the next day and new media collected the following day. We centrifuged the media at 3000×g for 15 min to pellet cell debris. We took the supernatant and filtered through a Millex-HV 0.45 µM PVDF filter (Millipore) and transferred it to a PEG-it Virus Precipitation solution (System Biosciences) and refrigerated overnight. Twelve hours later we centrifuged the lentivector-containing particles at 1500×g for 5 minutes and removed all traces of fluid, taking care to not disturb the pellet. We resuspended the pellet in 100 µl of OPTI-MEM and snap-froze individual aliquots at −70 C.

In order to achieve efficient knockdown of endogenous DISC1 without cytotoxic effects (which we frequently observed in both NIH3T3 cells and striatal neurons expressing the tagged construct at high levels as estimated by GFP fluorescence intensity), we co-transduced with different viruses, one encoding the silencing shRNA alone and another including the shRNA-resistant replacement construct. This achieved efficient knockdown of endogenous DISC1 while allowing independent titration of recombinant DISC1-GFP expression. With our preparations of viral particles we achieved optimal rescue at a ratio of 5/1, with 5 representing lentivirus containing shRNA and 1 being the lentivirus encoding DISC1-GFP (or GFP as a negative control). This strategy resulted in >80% knockdown of endogenous DISC1 (estimated from densitometry of immunoblots, see *Results*) and uniform, low levels of expression of DISC1-GFP in 90–95% of neurons (assessed by visualization of GFP fluorescence in the transduced cell population).

### Fluorescence microscopy

Colocalization of DISC1-GFP with acetylated tubulin or adenylyl cyclase III (ACIII) was visualized with cells plated on nitric acid etched coverslips and then treated with poly-D-lysine (Sigma) overnight. Cells were fixed with 3.7% formaldehyde and permeabilized with 0.1% Triton X- 100, and 3% milk in PBS. We incubated cells with mouse anti acetylated tubulin (Sigma, 1 µg/ml for 60 min) and rabbit anti adenylyl cyclase III-C20 (Santa Cruz Biotech, 0.8 µg/ml for 60 min), and then probed with goat anti rabbit Alexa594 (Invitrogen) and goat anti mouse Alexa488 (Invitrogen) respectively for 20 minutes. DISC1-GFP was localized relative to PCM1 using a rabbit antibody (H262, Santa Cruz Biotech, sc-67204, 1∶200 for 60 min) that has been previously validated [45]. In triple localization with mouse anti acetylated tubulin, detection of each protein was accomplished using goat anti rabbit Alexa594 and goat anti mouse Alexa647 (both from Invitrogen).

For cilia counts in NIH3T3 cells and primary striatal neurons, we stained for acetylated tubulin in NIH3T3 cells and ACIII in neurons as indicated above. Approximately 60% of NIH3T3 cells formed acetylated tubulin positive cilia in control conditions, and 40% formed ACIII positive cilia in primary dissociated striatal cultures. We arrived at the above numbers by counting the number of acetylated tubulin or ACIII positive cilia, respectively, and dividing it by the number of DAPI-positive cells. Each siRNA and short hairpin RNA condition was measured in the same manner. For each siRNA and short hairpin, experiments were conducted at least 5 times on separate days. In each experiment, ≥250 cells were examined for each condition.

Surface receptor immunoreactivity was assayed by incubating intact, non-permeabilized NIH3T3 cells with rabbit anti FLAG antibody (Sigma, 1 µg/ml for 15 min), washed, and fixed with 3.7% formaldehyde. Surface receptor immunoreactivity for non-permeabilized striatal primary dissociated neurons was assayed by incubating with mouse M1 anti-FLAG antibody (Sigma, 1 µg/ml for 15 min). Cells were washed and permeabilized with 0.1% Triton X- 100 in PBS, 3% milk, then incubated mouse anti acetylated tubulin (Sigma, 1 µg/ml for 60 min) or Rabbit anti AC III (Santa Cruz Biotech, 0.8 µg/ml for 60 min) followed by goat anti-rabbit Alexa594 and goat anti-mouse Alexa488 conjugate (Invitrogen) respectively.

All specimens were imaged by epifluorescence microscopy, using standard dichroic filter sets (Chroma) and a 60×, numerical aperture 1.4 objective (Nikon). Images were captured using a cooled CCD camera (Princeton Instruments) and exposures adjusted to avoid saturation. Acquired images were rendered with Adobe Photoshop software using linear lookup tables.

### Biochemical methods

Cell monolayers were washed three times in ice-cold phosphate-buffered saline (PBS) and lysed in extraction buffer (0.1% Triton X- 100, 150 mM NaCl, 25 mM KCl, 25 mM Tris, pH 7.4) supplemented with a standard protease inhibitor mixture (Roche Applied Science). Extracts were clarified by centrifugation (20,000×g for 15 min) and then mixed with lithium dodecylsulfate (LDS) sample buffer for denaturation and 1% β-mercaptoethanol for reduction, and incubated for 5 minutes at room temperature. Total protein levels for each well were normalized to each other by averaging 3 measurements of Coomassie Plus in a 96 well plate reader. Proteins present in the extracts were resolved by LDS-PAGE using 4–12% BisTris gels (NuPAGE; Invitrogen), transferred to nitrocellulose membranes, and probed for tagged protein by immunoblotting using the indicated primary antibody. Horseradish peroxidase-conjugated donkey anti-rabbit IgG (Amersham Biosciences) was used as secondary antibody, as appropriate, followed by detection of immunoreactivity using SuperSignal detection reagent (Pierce). Apparent molecular mass was estimated using commercial protein standards (Novex Sharp prestained protein standard, Invitrogen). Band intensities of unsaturated immunoblots were analyzed and quantified by densitometry using FluorChem 2.0 software (AlphaInnotech Corp.). Antibodies used were Rabbit-DISC1 (midi and C-term; Invitrogen catalog number 40–6900 and 40–6800, respectively; used at 1.5 µg/ml overnight), Rabbit anti IFT88 (Proteintech; used at 1/1000). The midi antibody was raised to a proprietary peptide immunogen corresponding to a middle portion (between residues 400 and 450) of NP_777279.1. The C-term antibody was raised to a proprietary peptide between residues 520 and 570 of NP_777279.1. Both antibodies have been previously characterized [46], [47].

### Statistical analysis

Quantitative data were averaged across multiple independent experiments, with the number of experiments specified in the corresponding figure legend. Unless indicated otherwise, the error bars represent the S.E.M. calculated across experiments. The statistical significance of the indicated differences was analyzed using Student's t test, calculated using Prism 4.0 software (GraphPad Software, Inc.).
